# Supplementary material for: What Are the Psycho-Social and Information Needs of Adolescents and Young Adults Cancer Care Consumers with Intellectual Disability? A Systematic Review of Evidence with Recommendations for Future Research and Practice
Source: Children (Basel). 2021 Dec 2;8(12):1118. doi: 10.3390/children8121118 (PMC8699987; doi:10.3390/children8121118)
Supplement: Supplementary file 1 [file children-08-01118-s001.zip › Children-1409409-supplementary.pdf]

Supplementally File 1

Key search terms were:

Intellectual Disabilit*.mp. or exp Intellectual Disability/ or Developmental Disabilit*.mp. or exp child development disorders, pervasive/ or (developmental* adj delay*).mp. or (developmental adj disorder*).mp. or Mentally Disabled Persons/
and (exp neoplasms/ or cancer.mp.)
and Psychosocial Support System*.mp
and (Adolescent* or Young Adult* or teenage* or AYA or adolescence).mp.

The full search strategy is detailed in Table S1 and Supplementary File S2

Supplementally File 2

Table S1 Search Strategy Summary

#	Words Searches
1	Intellectual Disabilit*.mp. or exp Intellectual Disability/ or Developmental Disabilit*.mp. or exp child development disorders, pervasive/ or (developmental* adj delay*).mp. or (developmental adj disorder*).mp. or Mentally Disabled Persons/
2	1 and (exp neoplasms/ or cancer.mp.)
3	2 and psycho*.mp.
4	2 and needs assessment*.mp.
5	2 and Psychosocial Support System*.mp
6	2 and stress, psychological/
7	2 and social support.mp
8	2 and Adaptation, Psychological.mp.
9	2 and Self-Help Groups.mp
10	2 and social.mp.
11	3 or 4 or 6 or 7 or 8 or 9 or 10
12	11 and (Adolescent* or Young Adult* or teenage* or AYA or adolescence).mp.
13	psychosocial intervention*.mp.
14	social support/
15	(social network* or social support or support system or support systems).mp. or self help groups/ or self help group.mp. or self help groups.mp.
16	(support group or support groups or educational therapy).mp. or psychotherapy/ or psychotherapy.mp. or behavior therapy.mp. or behaviour therapy.mp. or family therapy.mp. or group therapy.mp. or group interventions.mp. or cognitive therapy.mp.

	or cognition therapy.mp. or adaptation, psychological/ or psychological adjustment.mp. or psychological adjustments.mp. or psychological adaptation.mp. or adaptive behavior.mp. or adaptive behaviors.mp. or adaptive behaviour.mp. or adaptive behaviours.mp. or coping behavio*.mp. or coping intervention*.mp. or coping strateg*.mp. or coping skill*.mp. or (support adj2 need*).mp.
17	13 or 14 or 15 or 16
18	2 and 17
19	11 or 18
20	19 and (Adolescent* or Young Adult* or teenage* or AYA or adolescence).mp.
21	limit 20 to english language

Supplementally File 3

Electronic databases

- Medline
- Embase
- Emcare
- PsycINFO
- CINAHL
- Web of Science;
- Scopus
- Cochrane library

Grey literature-Handsearching

- Websites
    1. Agency for Clinical Innovation (ID Network)-Searched ID resource on the 6/3/2021—81 resources screened- 0 relevant: https://aci.health.nsw.gov.au/networks/intellectual-disability/resources
    2. Down Syndrome Australia -searched on the 6/3/2021 “cancer”- zero results: https://www.downsyndrome.org.au/?s=cancer
    3. Paediatric International Patient Safety and Quality Community (PIPSQC) - searched ‘Intellectual disability’ on the 6/3/2021-9 -0 relevant https://www.pipsqc.org/Search-Results?search=Intellectual%20disability\
    4. Australian Commission on Safety and Quality in Health Care on the 6/3/2021 -searched ‘Intellectual disability’ 6 resources -0 relevant https://www.safetyandquality.gov.au/search?keys=Intellectual+disability

5. Cancer Research UK-Searched on the 6/3/2021 searched ‘Intellectual disability’ zero results: https://find.cancerresearchuk.org/?xss-q=Intellectual+disability

6. MacMillan Cancer UK-Searched on the 15/3/2021 ‘Intellectual disability’ two results-0 relevant. https://www.macmillan.org.uk/search/search.html?query=Intellectual+disability
7. Canadian Partnership Against Cancer (CPAC): Searched on the 15/3/2021 ‘Intellectual disability’ seven results-0 relevant.
8. https://www.partnershipagainstcancer.ca/?s=Intellectual+disability&post_type=all&search_type=global#
9. Open Grey-Searched on the 6/3/2021- Intellectual disability-91 search-all screened -0 relevant http://www.opengrey.eu/search/request?q=Intellectual+disability
10. Princess Margaret Cancer Centre Canada Searched on the 15/3/2021 ‘Intellectual disability’ 15 results-0 relevant. https://www.cbcn.ca/en/search?search=Intellectual+disability

Key Terms: Intellectual Disability, Psychosocial Support, Cancer, young people

Journals for Hand Searching

- 294 articles table and abstract screen -13 screened for full text review

- Psycho-Oncology-Searched On the 30/3/21 Searched ‘Intellectual Disability” Search from 1/1/2000-12/3/2021
  101 results- 3 relevant
  https://onlinelibrary.wiley.com/action/doSearch?field1=AllField&text1=Intellectual+Disability&publication%5B%5D=10991611&Ppub=&AfterMonth=1&AfterYear=2000&BeforeMonth=3&BeforeYear=2021

- European Journal of Oncology Nursing- Searched On the 15/3/2021-Search from 1/1/2000-12/3/2021 Intellectual disability + young people-25 results-screened by ÉNS 1 relevant
  https://www.ejoncologynursing.com/action/doSearch?text1=Intellectual+disability&field1=AllField&text2=Young+People&field2=AllField&Ppub=&Ppub=&AfterMonth=1&AfterYear=2000&BeforeMonth=3&BeforeYear=2021&journalCode=yejon

- Cancer Medicine- Searched On the 15/3/2021-Search from 1/1/2000-12/3/20201 Intellectual disability + young people-2 Results-Screened by ÉNS 0 relevant
  https://onlinelibrary-wiley-com.wwwproxy1.library.unsw.edu.au/action/doSearch?AfterMonth=1&AfterYear=2000&BeforeMonth=3&BeforeYear=2021&Ppub=&field1=AllField&field2=AllField&field3=AllField&publication%5B%5D=20457634&text1=Intellectual+disability&text2=Young+People&text3=

- Journal of Intellectual Disability Research - Searched On the 15/3/2021-Search from 1/1/2000-12/3/20201 Cancer + young people-81 Results- majority on cancer incidence amongst people with ID: Screened by ÉNS 1 relevant

  https://onlinelibrary-wiley-com.wwwproxy1.library.unsw.edu.au/action/doSearch?field1=AllField&text1=cancer&field2=AllField&text2=Young+People&field3=AllField&text3=&publication%5B%5D=13652788&Ppub=&AfterMonth=1&AfterYear=2000&BeforeMonth=3&BeforeYear=2021

- Journal of Applied Research in Intellectual Disabilities- Searched On the 15/3/2021-Search from 1/1/2000-12/3/20201 Cancer + young people-75 Results- majority on cancer incidence amongst people with ID: Screened by ÉNS 8 relevant

  https://onlinelibrary-wiley-com.wwwproxy1.library.unsw.edu.au/action/doSearch?field1=AllField&text1=Cancer&field2=AllField&text2=Young+People&field3=AllField&text3=&publication%5B%5D=14683148&Ppub=&AfterMonth=1&AfterYear=2000&BeforeMonth=3&BeforeYear=2021

- Journal of Child Health Care- Searched On the 16/3/2021-Search from 1/1/2000-12/3/20201 Cancer + Intellectual Disability 8 Results Screened by ÉNS 0 relevant
- https://journals.sagepub.com/action/doSearch?field1=AllField&text1=Intellectual+Disability+&field2=AllField&text2=Cancer&publication%5B%5D=chca&Ppub=&Ppub=&AfterYear=2000&BeforeYear=2021&earlycite=on&access=

- Journal of Adolescent and Young Adult Oncology Searched On the 16/3/2021-Search from 1/1/2000-12/3/20201 searched “Intellectual Disability” 2 Results Screened by ÉNS 0 relevant
- https://www.liebertpub.com/action/doSearch?SeriesKey=jayao&AllField=Intellectual+Disability
